# Retesting and repeat positivity following diagnosis of *Chlamydia trachomatis* and *Neisseria gonorrhoea* in New Zealand: a retrospective cohort study

**DOI:** 10.1186/s12879-017-2635-y

**Published:** 2017-07-28

**Authors:** Sally B. Rose, Susan M. Garrett, James Stanley, Susan R. H. Pullon

**Affiliations:** 1 Department of Primary Health Care and General Practice, University of Otago, Wellington, PO Box 7343, Wellington South, 6242 New Zealand; 2 Biostatistical Group, University of Otago, Wellington, P O Box 7343, Wellington South, 6242 New Zealand

**Keywords:** *Chlamydia trachomatis*, *Neisseria gonorrhoea*, Sexually transmitted infections, Partner notification, Test of reinfection

## Abstract

**Background:**

Testing for reinfection at 3 to 6 months following treatment for *Chlamydia Trachomatis* or *Neisseria gonorrhoea* is recommended in best practice sexual health management guidelines. This study aimed to describe rates of retesting and repeat positivity following diagnosis of chlamydia or gonorrhoea in a defined geographic region of New Zealand.

**Methods:**

Retrospective cohort study in Wellington, New Zealand involving analysis of laboratory data for chlamydia and gonorrhoea tests performed in primary care and sexual health clinics (July 2012–July 2015). Outcome measures: rate of retesting and rate of repeat positivity 6 weeks to 6 months after a positive result (index event). Kaplan-Meier curves were used to plot time from first index event to retest. Logistic regression modelling was used to determine the odds of retesting and repeat positivity between 6 weeks and 6 months of follow-up, adjusting for potential confounders (age, gender, ethnicity and socioeconomic deprivation).

**Results:**

Overall 29.4% (1919/6530) of the cohort was retested between 6 weeks and 6 months, with 18% (347/1919) of those retested returning positive results. Lower odds of retesting were observed for males (OR 0.4, 95% CI 0.34–0.48), and individuals of NZ Maori (OR 0.72, 0.61–0.85) and Pacific ethnicities (OR 0.49, 0.39–0.62, reference European). Factors associated with higher odds of repeat positivity on retesting included male gender (OR 2.0, 1.14–2.82), age 15–19 years (OR 1.78, 1.32–2.41, reference 20–24 years), chlamydia/gonorrhoea co-infection (OR 2.39, 1.32–4.35, reference chlamydia only), Maori (OR 1.6, 1.18–2.17) and Pacific ethnicities (OR 1.88, 1.22–2.9, reference European).

**Conclusions:**

We observed low adherence to STI retesting guidelines, and marked gender and ethnic disparities in rates of retesting and repeat positivity. Low retesting rates are suggestive of low levels of awareness of this aspect of patient management, and an absence of a systematic approach to retesting. High rates of repeat positivity reinforce the importance of advising patients about reducing their risk of reinfection, including notification and treatment of all recent sexual partners. Greater priority needs to be placed on increasing retesting and reducing rates of reinfection, with strategies implemented to improve these important aspects of patient care and population STI control.

## Background

Individuals diagnosed with *Chlamydia trachomatis* or *Neisseria gonorrhoea* are a known risk group for subsequent reinfection [1]. Chlamydia remains the most commonly diagnosed bacterial sexually transmitted infection (STI) worldwide [2], despite the widespread availability of highly sensitive diagnostic technologies and highly effective single dose treatment for uncomplicated infection. Modelling studies suggest reinfection is likely to play a significant role in sustaining the global chlamydia epidemic [3], in turn highlighting the critical role that treating sexual partners plays in STI control.

Chlamydial infection occasionally persists due to treatment failure, but repeat positivity upon retesting is most often due to reinfection from an untreated sexual partner or an infected new partner [4, 5]. Undetected and untreated repeat infection can result in more serious long-term reproductive sequelae than an initial infection, with an estimated four-fold increased risk of pelvic inflammatory disease, a two-fold risk of ectopic pregnancy and a resultant higher risk of infertility [1, 5]. Treating partners together with early detection and treatment of reinfection are therefore important to minimize harm and onward transmission [6].

Testing for reinfection (rather than test of cure) at 3 months following treatment for both chlamydia and gonorrhoea is recommended in STI management guidelines [5, 7, 8]. If retesting at 3 months is not possible, some guidelines recommend retesting whenever care is next sought in the 12 months following treatment [5]. A test of cure is no longer routinely recommended for these bacterial infections due to the high efficacy of first line treatments, but is advised in more limited circumstances including pregnancy, when second line treatments were used, treatment compliance is in question, or symptoms persist [5, 9, 10]. Testing sooner than three to 5 weeks post-treatment for chlamydia or gonorrhoea is not recommended to avoid detection of residual nonviable organisms that can give a false-positive result [5, 7].

New Zealand has comparatively high rates of chlamydia with the burden of infection carried by young people (under 25 years), and particularly young Maori (the indigenous people of New Zealand) and Pacific peoples [11]. In 2014, chlamydia rates in New Zealand were 2092 and 3062 per 100,000 population for ages 15–19 and 20–24 respectively [11]. By comparison, 2014 chlamydia rates in the US were 1804 and 2485 per 100,000 for ages 15–19 and 20–24 [12], and in the UK, rates were 1708 and 2189 per 100,000 population for the same age-groups [13]. While there is no national screening programme in New Zealand, opportunistic chlamydia screening is recommended for females under 25 years meeting various risk criteria, and others deemed ‘at risk’ (e.g. two or more partners in the past 12 months, a recent partner change or inconsistent use of condoms) [9, 10]. Partner notification is recommended as part of chlamydia and gonorrhoea management using ‘patient referral’ as the preferred method, whereby patients are advised of the need to notify their sexual contacts of the past 2-months. If patient safety is a concern, ‘provider referral’ should be offered (the clinician notifies contacts with the patient’s consent) [9, 10]. New Zealand Sexual Health Society guidelines also recommend testing for reinfection at 3 months [9], and Ministry of Health chlamydia management guidelines recommend testing within three to 6 months [10]. The present study was designed to assess the extent to which guideline recommendations regarding retesting are met in a defined geographical region of New Zealand. Retesting rates within 6 months of a chlamydia or gonorrhoea diagnosis in primary care and sexual health clinics were analysed and demographic characteristics (age, ethnicity, gender) assessed to identify factors associated with receipt of retesting and repeat positivity.

## Methods

This study was reviewed and approved by the Southern Health and Disability Ethics Committee, New Zealand on July 16th 2015 (Ref 15/STH/109).

### Design and setting

This retrospective cohort study analysed data obtained from one diagnositic laboratory pertaining to chlamydia and gonorrhoea tests requested by primary care and sexual health service providers in the greater Wellington region of New Zealand (population approximately 450,000). The laboratory provides all community testing services for the region and uses the commercially available Roche cobas 4800 CT/NG assay [14] to detect *Chlamydia trachomatis* or *Neisseria gonorrhoea* from a single swab or urine specimen (specimens are routinely tested for both bacteria).

### Data collection

Laboratory data were obtained for tests performed in the 3 years from 1 July 2012 to 1 July 2015. The first positive chlamydia or gonorrhoea result for an individual occurring in the first two and a half years of data collection (1 July 2012 to 31 Dec 2014) was designated as the first ‘index event’ for that person, with follow-up to 1 July 2015 ensuring each person had a minimum of 6 months follow up (maximum 3 years). Exclusion criteria included: index events requested in secondary care (hospitals and specialists) with the exception of sexual health specialists; and tests performed for 0–9 year-olds (16 positives removed, all of which were neonatal eye infections). A small number of individuals with missing age and/or gender data were also excluded prior to analysis.

The dataset included laboratory number (assigned by the lab to unique individuals), National Health Index NHI (a unique patient identifier assigned to all New Zealanders at birth that enables health record linkage) [15], gender, date of birth, visit date, type of specimen, result, requestor name (Dr, nurse), requesting location (clinic). No patient names, addresses or contact details were obtained. Prioritized ethnicity and NZDeprivation06 (NZDep06) scores were obtained via NHI matching from nationally held datasets. Ethnicity refers to the ethnic group with which an individual self-identifies and is collected via self-report using the standardized New Zealand 2001 census question [16]. NZDep06 is a validated, census-derived, area-based index of socioeconomic deprivation, grouped into deciles 1 to 10, where 1 represents least deprived areas and 10 most deprived areas [17]. NHI and gender are not mandatory fields for laboratory requests so are not routinely provided by some requestors, and therefore ethnicity and NZDep could not be ascertained for some individuals.

### Data processing and identification of cohort for analysis

SAS Enterprise Guide (v7.1) was used to clean and format data. Duplicate records were removed where multiple specimens were tested for one individual on the same day, or when retesting occurred within a few days of the index event due to inadequate specimen collection. Unique laboratory numbers were used to identify multiple tests for a given individual, and a single line of data created for each test-retest event. A test undertaken at least 6 weeks (42 days) after the index event was considered a ‘retest’ (outcome 1) and examined as to whether the retest was positive for chlamydia or gonorrhoea (outcome 2). Retesting soon after treatment is more likely to have been performed as ‘test of cure’ and carries with it a higher likelihood of false positives. Any repeat positives within 42 days of diagnosis were not therefore considered new infections in this analysis (assumes a median time to treatment of 6 days [18], plus 5 weeks).

Each person could contribute more than one index event test sequence to the dataset. If the retest following an index event was negative, that marked the end of the test-retest sequence; however any subsequent positive test (>42 days later) for that person became a new index event which was followed-up for retesting outcomes using the protocol above.

### Statistical analyses

Proportions of the overall cohort testing positive for chlamydia and gonorrhoea infection were described with respect to gender, age and ethnicity. Kaplan-Meier curves were used to depict time from first index test to retest by gender (to a maximum of 2 years follow-up). All individuals included in this analysis had a minimum of 6 months follow-up, 80% had at least 12 months and 39% had at least 24 months follow-up.

Outcome measures were i) proportion of individuals retested for chlamydia and gonorrhoea 6 weeks to 6 months after a positive test (described by age, sex, ethnicity, NZDep); and ii) the proportion of retested individuals with positive results for chlamydia/gonorrhoea (described by age, sex, ethnicity, NZDep). Logistic regression models were used to examine associations between the outcomes and exposure variables (type of infection at index presentation, age, sex, ethnicity, NZDep). These models explicitly allowed for individuals to have multiple index events in the analysis by accounting for the non-independence of observations that arises when some individuals have more than one index event (using a generalized linear mixed model with a binomial link function and random intercepts to account for correlation between multiple index events from the same individual). Separate analyses were conducted for retesting between 6 weeks and 6 months of an index event (outcome one: denominator is index events), and for having a positive test on retesting within this timeframe (outcome two: denominator is all retests). Statistical analyses were conducted using Stata 12 (StataCorp, Tx).

## Results

### Description of the study cohort

**Fig. 1:**
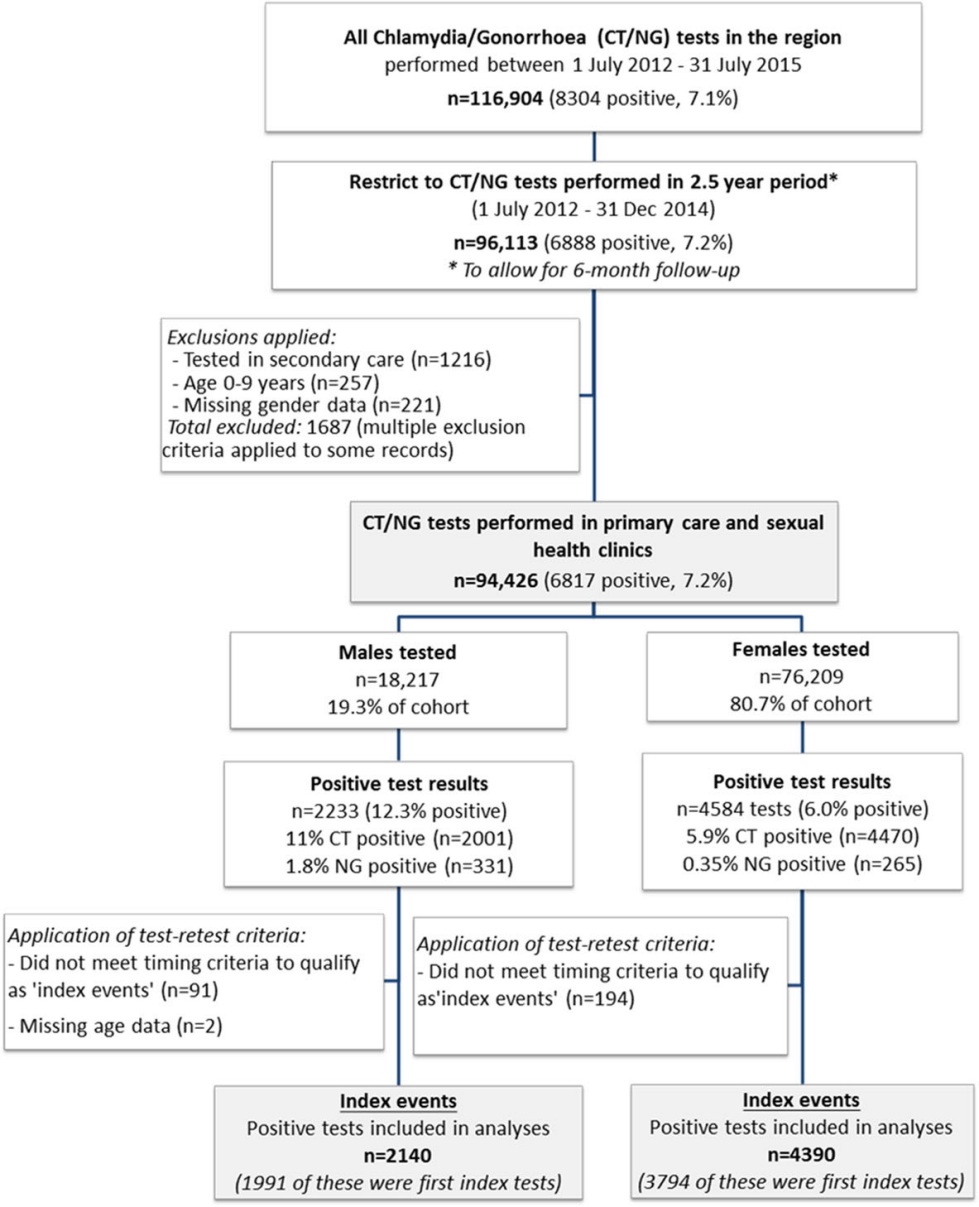
Process by which index events (positive tests) were selected for inclusion in outcome analyses

Figure 1 depicts the process by which index events (positive tests) were selected for inclusion in outcome analyses. During the two-and-a-half-year period, a total of 94,426 tests were performed in primary care and sexual health clinics (18,217 for males and 76,209 for females) with 6817 testing positive overall (7.2% positivity). There were clear differences in rates of positivity by gender, age and ethnicity for the cohort tested. Males made up only 19.3% of those tested, but were more likely than females to test positive for chlamydia (11% versus 5.9%) and gonorrhoea (1.8% versus 0.4%). The majority of those testing positive were aged under 25 years (65%). In this age group (detail not included in Fig. 1), 16.1% of males and 9.5% of females were diagnosed with chlamydia, and 5.7% of males and 0.6% of females diagnosed with gonorrhoea. Individuals of Pacific ethnicities had the highest proportion of positive results (16.3%), followed by Maori (14.1%), European (5.2%) and Asian (5.1%). Following application of test-retest criteria (and removal of two cases with no age data), 6530 positive index events were included in the dataset for further analysis (2140 index tests for males, 4390 index tests for females).

### Repeat testing

Table 1 presents the unadjusted data for 6530 index events for individuals who tested positive during the study period (*n* = 5785) and the number and percentage who were then a) retested between 6 weeks and 6 months of a chlamydia or gonorrhoea diagnosis, and b) diagnosed with a positive result on retest. During this period, 29.4% (1919/6530) of the cohort were retested for chlamydia and gonorrhoea within the recommended 6 month period, of whom 18% (347/1919) tested positive. Unadjusted retesting and repeat positivity rates varied by age, gender, ethnicity and index test location. Gender differences were observed in proportions retested within the recommended timeframe, with 35% of females retested (95% CI 33.6–36.4) but only 17.9% of males (95% CI 16.3–19.6). When retested, 21.1% of males had repeat positives (95% CI 17.2–25.6) compared to 17.3% of females (95% CI 15.5–19.3).

**Table 1:** Characteristics of the cohort with index positive tests (*n* = 6530), proportions retested and positive on retesting

Patient characteristics^a^	Males					Females				
	Index Positive	Retested 6wks-6mths		Positive on retest		Index Positive	Retested 6wks-6mths		Positive on retest	
	*n*	*n*	(%)	*n*	(%)	*n*	*n*	(%)	*n*	(%)
Total	2140	383	(17.9)	81	(21.1)	4390	1536	(35.0)	266	(17.3)
Index infection										
Chlamydia	1825	330	(18.1)	67	(20.3)	4139	1454	(35.1)	245	(17.5)
Gonorrhoea	220	44	(20.0)	9	(20.5)	105	30	(28.6)	3	(10.0)
Both	95	9	(9.5)	5	(55.6)	146	52	(35.6)	18	(34.6)
Age-band										
10–14	0	-^b^	−	−	−	39	18	(46.2)	6	(33.3)
15–19	301	40	(13.3)	14	(35.0)	1277	524	(41.0)	129	(24.6)
20–24	842	157	(18.6)	39	(24.8)	1840	646	(35.1)	96	(14.9)
25–29	422	66	(15.6)	9	(13.6)	673	206	(30.6)	19	(9.2)
30–34	209	37	(17.7)	5	(13.5)	292	82	(28.1)	12	(14.6)
35–39	131	25	(19.1)	3	(12.0)	129	31	(24.0)	3	(9.7)
40–44	96	17	(17.7)	4	(23.5)	71	13	(18.3)	1	(7.7)
45–49	62	16	(25.8)	2	(12.5)	42	9	(21.4)	0	(0.0)
50–54	41	14	(34.1)	4	(28.6)	17	6	(35.3)	0	(0.0)
55–59	15	2	(13.3)	1	(50.0)	5	1	(20.0)	0	(0.0)
60+	21	9	(42.9)	0	(0.0)	5	0	(0.0)	−	−
Ethnicity										
European	951	201	(21.1)	36	(17.9)	1817	716	(39.4)	95	(13.3)
NZ Maori	440	62	(14.1)	21	(33.9)	1485	533	(35.9)	113	(21.2)
Pacific	245	34	(13.9)	9	(26.5)	618	163	(26.4)	37	(22.7)
Asian	105	31	(29.5)	3	(9.7)	221	80	(36.2)	15	(18.8)
MELAA/Other^c^	42	12	(28.6)	3	(25.0)	47	22	(46.8)	4	(18.2)
Not known	357	43	(12.0)	9	(20.9)	202	22	(10.9)	2	(9.1)
Socioeconomic deprivation (NZDep)										
Least deprived (1–3)	425	77	(18.1)	14	(18.2)	773	290	(37.5)	40	(13.8)
Moderately dep (4–7)	538	94	(17.5)	19	(20.2)	1362	522	(38.3)	83	(15.9)
Most deprived (8–10)	730	157	(21.5)	37	(23.6)	1981	689	(34.8)	140	(20.3)
Not known	447	55	(12.3)	11	(20.0)	274	35	(12.8)	3	(8.6)
Test location/clinic^d^										
General Practice	913	142	(15.6)	37	(26.1)	2069	606	(29.3)	109	(18.0)
Sexual Health	604	134	(22.2)	20	(14.9)	208	45	(21.6)	3	(6.7)
Student/Youth	323	59	(18.3)	17	(28.8)	999	434	(43.4)	80	(18.4)
Family Planning	300	48	(16.0)	7	(14.6)	1114	451	(40.5)	74	(16.4)

^a^The dataset includes 5785 unique individuals, some with multiple index tests

^b^The - sign indicates cells with no information (either no index tests in group, or no retests performed and reinfection frequency is zero by definition)

^c^MELAA is an ethnic grouping that includes Middle Eastern, Latin American and African

^d^Includes 112 individual locations/clinics

### Time to retesting

**Fig. 2:**
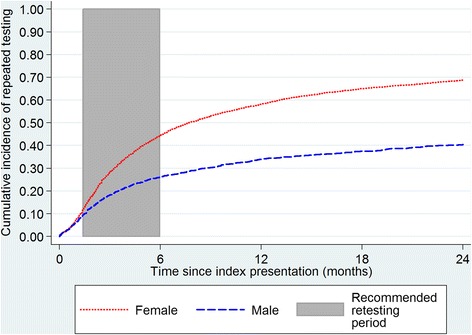
Kaplan-Meier curves for time from first index event to subsequent test during two year follow-up

Figure 2 presents Kaplan-Meier curves showing time from first index event to retesting for 5785 males and females (recommended retesting period is highlighted in grey). Within 6 weeks of the first index positive, 11.6% of females (440/3794) and 9.3% of males (186/1991) had been retested, of whom 22.0% (97/440) of females and 29.0% (54/186) of males retested positive. By 6 months, 44.5% of females and 26.2% of males had been retested (including tests within 6 weeks of index events). Gender differences persisted over time, with 58.2% of females and only 34% of males retested within 0–12 months; and 68.8% of females and 40.5% of males retested within 0–24 months.

### Correlates of retesting and repeat positivity

Table 2 presents the odds ratios and confidence intervals from logistic regression analyses (generalized linear mixed models) examining factors independently associated with retesting and repeat positivity (separate analyses) between 6 weeks and 6 months of a positive chlamydia or gonorrhoea index event. Factors associated with significantly lower odds of retesting included male gender (OR 0.4, 95% CI 0.34–0.48, reference female), NZ Maori ethnicity (OR 0.72, 0.61–0.85, reference European) and Pacific ethnicity (OR 0.49, 0.39–0.62, reference European). Factors associated with significantly higher odds of repeat positivity when retested included male gender (OR 2.0, 1.41–2.81), younger age (for ages 15–19, OR 1.78, 1.32–2.41, reference 20–24), co-infection with both chlamydia and gonorrhoea at the index event (OR 2.39, 1.32–4.35, reference chlamydia only), and NZ Maori (OR 1.6, 1.18–2.17, reference European) and Pacific ethnicities (OR1.88, 1.22–2.9, reference European).

**Table 2:** Logistic regression analyses for factors associated with retesting for chlamydia/gonorrhoea and repeat positivity on retesting

Patient characteristics^a^	Retested at 6-weeks to 6-months (*n* = 6530)			Repeat positive on retesting (*n* = 1919)		
	Odds Ratio	(95% CI)	Factor *p*-value	Odds Ratio	(95% CI)	Factor *p*-value
Gender			<0.001			<0.001
Male	0.40	(0.34, 0.48)		2.00	(1.41, 2.82)	
Female	Reference			Reference		
Index infection						
Chlamydia	Reference		0.593	Reference		0.011
Gonorrhoea	1.00	(0.72, 1.39)		0.76	(0.38, 1.52)	
Both	0.83	(0.58, 1.19)		2.39	(1.32, 4.35)	
Age-band						
10–14	1.77	(0.80, 3.94)		2.60	(0.86, 7.85)	
15–19	1.23	(1.04, 1.46)		1.78	(1.32, 2.41)	
20–24	Reference		0.001	Reference		<0.001
25–29	0.83	(0.68, 1.01)		0.53	(0.33, 0.84)	
30–34	0.79	(0.60, 1.04)		0.71	(0.39, 1.27)	
35–39	0.69	(0.48, 1.01)		0.54	(0.22, 1.37)	
40+	0.91	(0.67, 1.25)		0.61	(0.30, 1.23)	
Ethnicity						
European	Reference		<0.001	Reference		0.018
NZ Maori	0.72	(0.61, 0.85)		1.60	(1.18, 2.17)	
Pacific	0.49	(0.39, 0.62)		1.88	(1.22, 2.90)	
Asian	1.12	(0.83, 1.52)		1.38	(0.77, 2.47)	
MELAA/Other^b^	1.62	(0.93, 2.84)		1.50	(0.59, 3.82)	
Not known	0.68	(0.40, 1.15)		2.33	(0.58, 9.43)	
Socioeconomic deprivation (NZDep)						
Least deprived (1–3)	Reference		<0.001	Reference		0.226
Moderately dep (4–7)	1.10	(0.90, 1.33)		1.11	(0.75, 1.63)	
Most deprived (8–10)	1.11	(0.92, 1.35)		1.35	(0.93, 1.96)	
Not known	0.43	(0.27, 0.69)		0.61	(0.17, 2.15)	

^a^The dataset includes 5785 unique individuals, some with multiple index tests

^b^MELAA is an ethnic grouping that includes Middle Eastern, Latin American and African

## Discussion

This analysis suggests many clinicians are not following guideline recommendations to routinely test for reinfection at three to 6 months. Marked gender and ethnic disparities were observed both in retesting and reinfection rates. Males, Maori and Pacific ethnic groups were less likely to be retested within the recommended period, but when retested, these groups were among those most likely to return positive results. Overall rates of repeat positivity were high among those retested, with a quarter of females and a third of males aged 15–19 years testing positive, a finding likely to be indicative of low levels of partner treatment. Younger age groups (24 years and under) were more likely to be retested than those aged 25 years and older, in line with known risk profiles, but retesting rates were still low, particularly for young males. Co-infection with chlamydia and gonorrhoea was a risk factor for having a subsequent positive retest result, yet individuals in this group were tested no more frequently than those with a single-bacterial infection.

Whereas some studies reporting reinfection and retesting rates have focussed only specific patient populations (often limited to females) [4, 19, 20], a strength of our study was the robust sample comprising laboratory data for all males and females diagnosed with chlamydia or gonorrhoea in the region over a two-and-a-half-year period. Test locations included over 100 primary health care (general practice, youth health, student health and family planning) and sexual health clinics and all individuals had a minimum of 6 months follow-up. Limitations of the analysis included lack of behavioural and clinical data that would have helped in the interpretation of study findings (such as condom use, change of sexual partner, number and gender of recent partners, presence of symptoms, and treatment outcomes for index cases and their partners). While most of those with repeat positives will have represented new infections; a small proportion would have been persistent infection due to treatment failure or compliance issues (regardless, any repeat positive is important to pick up on retesting). Reasons for retesting were unknown, but would presumably include patient/clinician concerns about reinfection as well as opportunistic retesting as per guideline recommendations. We do not know what advice was given about reinfection or retesting, nor the extent to which individuals may have re-presented for care but not been offered retesting. Some cohort members will have moved during follow-up, and any retesting outside the study region will have been missed (hence underestimating retesting rates). Retesting may have been targeted towards those individuals identified as being at higher risk for reinfection, thus over-estimating the prevalence of reinfection in this cohort.

Only one other estimate of repeat testing rates has so far been published in New Zealand - approximately 32% of females and 16% of males were retested within 6 months of a positive chlamydia result in the Waikato region between 2008 and 2010 [18]. Our data were more recent and related to a different region but showed very similar levels of retesting. This suggests retesting may be falling well short of best practice guidelines throughout New Zealand – a finding that warrants urgent attention, particularly in light of our persistently high initial infection rates. Although proportions retested within 12 months in our study were good, retesting sooner – closer to 3 months - may be more likely to prevent the development of long-term reproductive health complications [21]. Our retesting rates were similar to rates reported in Australia, where 12% of heterosexual males and 18% of heterosexual females were retested in the 4 months following treatment for chlamydia in a study of sexual health services [22], and 14% of males and 29% of females were retested in a study based in general practice [23]. In the largest US study to date, laboratory data for over 3 million chlamydia tests showed that only 22% of males and 38% of non-pregnant females were retested within 12 months [24].

The high rate of repeat positivity observed in the current analysis was in line with reinfection rates reported previously [4, 20]. Estimated chlamydia reinfection rates among 16–24 year-old females recruited into a UK study were 22 per 100 person years for those treated in Family Planning clinics and 29.9 per 100 person years for those treated in general practice [20]. The incidence of reinfection among adolescent women attending primary care clinics in the US was 34 episodes per 100 woman-years of follow-up [4]. The high rates of repeat positivity observed here and in past studies reinforces the importance of timely access to retesting to detect and treat reinfection early.

Our analysis shows that greater effort needs to be put into ensuring equitable access to timely retesting (and initial testing) for males, Maori and Pacific groups in New Zealand. The lower rates of retesting for males in this study may reflect patterns of initial testing, where males made up only a fifth of the total cohort tested. Males present to primary care less frequently than females [25], and diagnostic testing is more likely to be for symptomatic infection or on presentation as a contact of a known case. By contrast, females more often receive opportunistic screening, typically when presenting to primary care for contraception or cervical smears. Pacific people and Maori had substantially lower odds than Europeans to receive retesting (OR 0.5 and 0.7 respectively), yet amongst those retested the odds of reinfection were over 60% higher among Maori and 88% higher among Pacific (compared to European). Ethnic disparities in STI rates are well documented in New Zealand and are indicative (at least in part) of issues relating to inequitable access to appropriate healthcare [26]. Cost and other access-related issues may be prohibitive to return visits for some. Results from the 2012/13 New Zealand Health Survey showed that that Maori (and to a lesser extent Pacific) were more likely than European to report recent unmet need for healthcare due to cost [25].

The relatively low rates of retesting and high rates of repeat positivity in this study are suggestive of low levels of clinician awareness of this important aspect of patient care, and a lack of a systematic approach to partner notification and retesting. This suggestion is supported by the findings of a survey conducted in the same region in which 121 primary care clinicians (doctors and nurses among those with the highest annual STI diagnosis rates in the region) were asked about their approaches to partner notification and testing for reinfection [27]. Results showed that while partner notification was typically discussed at the time of treatment, a range of challenges were perceived to limit their ability to effectively undertake this discussion and follow-up on notification outcomes was rare. The survey also highlighted confusion among participants regarding the need for ‘test of cure’ versus a ‘test of reinfection’, with the majority indicating that active recall for repeat testing was uncommon [27]. Patient-factors including lack of knowledge about retesting and continued exposure to untreated partners are also likely to be important factors contributing to our study findings that need addressing.

Strategies to improve re-attendance rates among sexual health clinic attendees have demonstrated moderate levels of success. In two Australian studies, retesting rates increased by 9 and 22% when a text (SMS) message was sent to remind patients about a 3-month test of reinfection [28, 29]. A similar study in the Netherlands demonstrated a 21% increase in retesting [30]. The provision of a postal home sample-collection kit (in addition to an SMS) further improved retesting rates (an additional 22% retested compared to SMS alone) in another Australian study [31]. Missed opportunities for retesting in general practice have been reported, with patients re-presenting but not receiving retesting [23], which highlights the potential role of electronic chart prompts/flags to facilitate the offer of opportunistic testing when patients next present for care [1, 23]. Encouraging patients to put a reminder in their mobile phone, or offering drop-in clinics that allow asymptomatic patients to provide a self-sample, have also been suggested as strategies that may facilitate retesting [32].

## Conclusions

This study highlights the need to raise awareness of retesting for chlamydia and gonorrhoea reinfection at 3 months post-treatment as an important aspect of best practice STI patient management. Provision of verbal advice and use of a proactive approach to retesting should be accompanied by reinfection risk reduction counselling that includes the importance of partner treatment, advice not to have sex for 7 days following completion of own and partners’ treatment, consistent condom use and behavioural change if relevant. Prioritizing strategies to detect and prevent reinfection among those diagnosed will be critical if we are to reduce the burden and sequelae of bacterial STIs in our communities.
